# Enhanced Mechanical Properties of PUMA/SiO_2_ Ceramic Composites via Digital Light Processing

**DOI:** 10.3390/polym16020193

**Published:** 2024-01-09

**Authors:** Jiwan Kang, Seong Hyeon Park, Keun Park

**Affiliations:** 1 Institute of 3D Printing Convergence Technology, Seoul National University of Science and Technology, Seoul 01811, Republic of Korea; jwkang@seoultech.ac.kr; 2 Material Research Center, Carima Co., Ltd., Seoul 07532, Republic of Korea; shpark@carima.co.kr; 3 Department of Mechanical System Design Engineering, Seoul National University of Science and Technology, Seoul 01811, Republic of Korea

**Keywords:** additive manufacturing, ceramic 3D printing, digital light processing (DLP), polymer-matrix composites (PMCs), silica composites

## Abstract

This study aims to enhance the mechanical properties of additively manufactured polymer parts by incorporating ceramic particles (SiO_2_) into diluted urethane methacrylate (UDMA) photopolymer resin using digital light processing (DLP) technology. The resulting PUMA/SiO_2_ composites, featuring varying SiO_2_ contents (16.7, 28.5, and 37.5 wt%) and processed under different conditions, underwent a comprehensive series of mechanical, thermal, and chemical tests. Hardness tests showed that composites with 37.5 wt% SiO_2_ demonstrated superior hardness with low sensitivity to processing conditions. Bending tests indicated that elevated vat temperatures tended to degrade flexural properties, yet this degradation was mitigated in the case of the 37.5 wt% SiO_2_ composition. Tensile tests revealed a transition from viscoelastic to linear elastic behaviors with increasing SiO_2_ content, with high tensile strength sustained at low vat temperatures (<35 °C) when the SiO_2_ content exceeded 28.5 wt%. Thermogravimetric analysis supported these findings, indicating that increased SiO_2_ content ensured a more uniform dispersion, enhancing mechanical properties consequently. Thermal tests showed augmented thermal conductivity and diffusivity with reduced specific heat in SiO_2_-inclusive composites. This study provides guidelines for optimal PUMA/SiO_2_ composite utilization that emphasizes high SiO_2_ content and low vat temperature, offering comprehensive insights for high-performance ceramic composite fabrication in functional applications.

## 1. Introduction

Ceramic materials offer versatile advantages, including high mechanical strength and rigidity, wear resistance, and thermal and chemical stability, as well as thermal and electrical insulation capabilities. Despite these valuable properties, the mechanical machining of ceramic components is challenging due to their extreme hardness and brittleness, limiting their ability to be fabricated into complex 3D shapes [1]. Instead, the manufacturing of ceramic components with complex geometries typically involves employing a combination of ceramic powders and liquid binders. The resulting ceramic feedstock mixture (slurry) is then shaped using conventional manufacturing technologies such as injection molding, compression molding, tape casting, etc. [2]. The formed green ceramic parts undergo debinding and sintering processes to remove binders and achieve fusing and densification of ceramic powders [3].

Recent advances in three-dimensional (3D) printing, also known as additive manufacturing (AM), have propelled the fabrication of ceramic components with complex 3D shapes [4]. Various AM technologies, including binder jetting (BJ), powder-bed fusion (PBF), material extrusion (ME), and vat-photopolymerization (VP) processes, have been employed in the fabrication of 3D ceramic components [5]. The BJ process selectively sprays a liquid binder solution onto a layer of ceramic powders through an array of microscale nozzles, building layerwise ceramic green parts [6,7,8,9]. The PBF process, also known as selective laser sintering, uses a high-power laser beam to selectively irradiate the surface of the target ceramic powders for selective heating and sintering [10,11,12,13]. The ME process, also referred to as fused deposition or fused-filament fabrication process, achieves AM by extruding a heated thermoplastic filament through a narrow nozzle [14]. This process has been employed in ceramic AM by preparing a composite filament that contains ceramic particles [15,16,17,18].

The VP-type AM method can be categorized into the stereolithography (SL) and digital light processing (DLP) processes based on the light source type [19]. These VP processes construct a 3D object by selectively irradiating UV light on liquid photopolymer resin. In the SL process, a UV laser is employed to scan the liquid resin in the vat along predefined laser paths [20]. This technique has been widely applied in the AM of ceramic components [21,22,23,24]. On the other hand, the DLP process uses a digital projector to irradiate UV light for a sectional image onto the liquid resin surface [25] and has also been utilized in the AM of various ceramic components [26,27,28].

Previous studies have demonstrated that the DLP process offers advantages in terms of high manufacturing speed as it cures the entire layer simultaneously [29], while the SL process requires a longer printing time to scan and irradiate the predetermined laser paths [30]. Capitalizing on this advantage, the DLP process has been recently employed in the additive manufacturing of versatile ceramic components, including microcellular structures [31,32,33,34,35] and biomedical applications [36,37]. These studies commonly incorporated the debinding and sintering processes following the AM process to transform green parts (i.e., polymer-ceramic composites) into pure ceramic components.

The primary objective of this study is to improve the mechanical properties of additively manufactured polymer-matrix composites through the incorporation of ceramic particles. Specifically, silica (SiO_2_) powders were integrated into diluted urethane methacrylate (UDMA) photopolymer resin to enhance mechanical performance. While prior research has addressed the enhancement of silica-based ceramic composites [21,38,39,40], those studies have predominantly focused on the SL-type AM process. In contrast, this study employs the DLP-type AM process for the rapid fabrication of ceramic composites, with a particular emphasis on exploring the associated processing conditions and their impact on mechanical and thermal properties.

The additively manufactured ceramic composites with varying SiO_2_ contents, denoted as PUMA/SiO_2_, then underwent comprehensive analyses to evaluate their mechanical and thermal properties. To assess the reinforcement effect on mechanical properties corresponding to the inclusion of SiO_2_, a series of mechanical tests encompassing hardness, bending, and tensile tests were systematically conducted. Additionally, a meticulous exploration of the alterations in thermal properties of the PUMA/SiO_2_ composites was carried out through thermal diffusivity tests and thermogravimetric analysis (TGA). Through comprehensive analyses of these test results, this study systematically investigates PUMA/SiO_2_ composites with varied SiO_2_ contents and processing conditions, elucidating the distinct effects of ceramic reinforcement in DLP-type AM and offering guidelines for optimal utilization of PUMA/SiO_2_ composites.

## 2. Materials and Methods

### 2.1. Materials

As a base resin, diluted urethane methacrylate (UDMA) photopolymer (CUKCS08, Carima Inc., Seoul, Republic of Korea) was used. The photocurable resin was formulated for deployment in a DLP-type AM, which was optimized for UV curing at a wavelength of 405 nm. A photoinitiator (TPO, Diphenyl(2,4,6-trimethylbenzoyl)phosphine oxide, IGM Resins, Waalwijk, The Netherlands) was introduced to facilitate appropriate photocuring. The resulting product after photocuring is denoted as polymerized urethane methacrylate (PUMA).

Spherical silica (SiO_2_) powders with silane surface treatment were employed as ceramic particles in this study. These ceramic powders have a median diameter of 1.5 µm and specific surface area of 3−5 m^2^/g. Their density, thermal diffusivity, and conductivity are 2.07 g/cm^3^, 0.71 J/g-K, and 1.43 W/m-K, respectively. The sintered part using the SiO_2_ powders is known to have a compressive strength of 325.2 MPa [41] and a flexural strength of 48.7 MPa [42].

**Figure 1:**
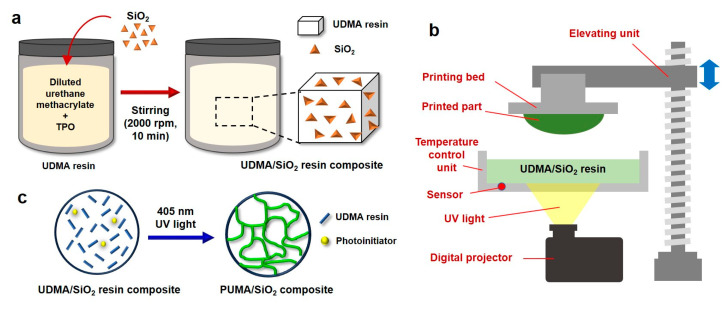
Additive manufacturing of PUMA/SiO_2_ composites: (**a**) preparation of UDMA/SiO_2_ composite resin, (**b**) DLP-type 3D printer equipped with a temperature-controlled vat, and (**c**) photopolymerization of PUMA/SiO_2_ composite.

In the preparation of ceramic slurry, SiO_2_ particles were blended with UDMA resin in varying weight ratios of 100:20, 100:40, and 100:60. Considering that the density of SiO_2_ particles is 2.07 g/cm^3^, the resulting weight fractions for the three weight ratios become 16.7 wt%, 28.5 wt%, and 37.5 wt%, respectively. The amalgamation of UDMA resin and varying ratios of SiO_2_ powder was carried out utilizing a Thinky defoam mixer (ARM-310, Thinky, Laguna Hills, CA, USA), as depicted in Figure 1a, operating at 2000 rpm for 10 min. Subsequently, three compositions of UDMA/SiO_2_ composite resins were prepared for the DLP-type AM process.

### 2.2. Additive Manufacturing

A DLP-type 3D printer (IMC, Carima Inc., Seoul, Republic of Korea) was utilized to additively manufacture PUMA/SiO_2_ composites. This printer is furnished with a UV lamp emitting light at a wavelength of 405 nm and is equipped with a high-definition digital micromirror device (DMD) chip boasting a resolution of 1920 × 1080 pixels. Notably, the resin vat is integrated with a temperature control unit, as depicted in Figure 1b. The layer thickness was configured at 50 μm, and the irradiation time per layer was set at 2.0 s. The vat temperature was varied within the range of 25 to 45 °C, with the reference temperature established at 35 °C.

The additively manufactured ceramic composites were subjected to a cleaning process, which involved the use of a solution of 70% ethanol and isopropanol (IPA) within an ultrasonic cleaning machine (Ultra 200H, Sinhan-Sonic Co., Incheon, Republic of Korea). Following the cleaning procedure, the composites underwent an additional curing step using a UV-curing machine (CL300Pro, Carima Inc., Republic of Korea) operating at a wavelength of 405 nm. During the postcuring process, the light density was adjusted to 110 mW/cm², and the curing time was varied within the range of 90 to 150 s, with the reference condition being set at 120 s. Following these AM and subsequent processes, the UDMA/SiO_2_ composite resin was fully polymerized, resulting in the fabrication of the PUMA/SiO_2_ composite, as illustrated in Figure 1c.

### 2.3. Rheological Test

Viscosities of four distinct UDMA/SiO_2_ composite resins, including UDMA, were quantified using a viscometer (DV-1M, Brookfield, WI, USA). The viscosity assessments were conducted at three different vat temperatures (25 °C, 35 °C, and 45 °C) employing a heated water bath on a hot plate equipped with a magnetic bar. For each temperature condition, viscosities were measured by incrementally varying the spindle speed up to 60 rpm.

### 2.4. Mechanical Tests

To access the mechanical properties of the additively manufactured PUMA/SiO_2_ composites across varying SiO_2_ concentrations and processing conditions, various mechanical tests were conducted including hardness, bending, and tensile tests. For the hardness test, Shore D hardness values of printed samples were measured using a Shore hardness tester (LXD-A, Wenzhou Sanhe Measuring Inc., Wenzhou, China). 

In the bending test, bending specimens with dimensions of 80 × 10 × 4 mm were designed following the ISO 178 standard [43]. The bending specimens were additively manufactured in a flat position, aligning the thickness direction with the z-direction. Bending tests were conducted using a universal test machine (NA-2M, Nanotech, Gimhae-si, Korea) at a deformation rate of 2 mm/min. The flexural modulus (*E_f_*) and strength (*σ_f_*) were calculated using the following equations:

(1) $$E_{f}=\frac{PL^{3}}{4bd^{3}v}\;$$

(2) $$\sigma_{f}=\frac{3PL}{2bd^{2}}\;$$

where *P* is the critical force, *L* is the span length, *b* is the beam width, *d* is the beam thickness, and *v* is the beam deflection.

For the tensile test, specimens were designed following the ASTM D638 type IV standard [44]. Similarly to the bending specimens, the tensile specimens were fabricated in a flat position. Tensile tests were performed using a universal test machine (NA-2M, Nanotech, Korea) at a deformation rate of 1 mm/min. These tests were systematically carried out on five specimens for various combinations of composite weight fractions and processing conditions.

### 2.5. Thermal Tests

To access the thermal properties of PUMA/SiO_2_ composites across varying SiO_2_ concentrations, thermal diffusivity tests were conducted using a laser flash analyzer (Netzsch LFA 467 HT, Netzsch-Gerätebau GmbH, Selb, Germany) following the ASTM E1461 standard [45]. The device is equipped with a xenon flash lamp, a liquid nitrogen-cooled InSb IR detector, and furnaces. The test samples were additively manufactured with a dimension of 10 × 10 × 1 mm, under the conditions of 35 °C vat temperature and 150 s postcuring. Through the thermal diffusivity tests, various thermal properties including thermal diffusivity (α), thermal conductivity (*k*), and specific heat (*C_p_*) were obtained. These properties are related by the following equation:

(3) $$\alpha =\frac{k}{\rho C_{p}}\;$$

where *ρ* is the bulk density of the PUMA/SiO_2_ composites.

To evaluate the thermal stability of PUMA/SiO_2_ composites, thermogravimetric analysis (TGA) was conducted. Samples with different SiO_2_ contents were prepared by cutting PUMA/SiO_2_ specimens into 10 mg pieces. The experiments were carried out using a thermal analysis system (DTG-60H, Shimadzu, Kyoto, Japan) with a heating rate of 10 °C/min under nitrogen conditions, spanning a temperature range from 30 °C to 550 °C. The flow rate of hot air was maintained at 60 mL/min.

### 2.6. Other Characterizations

The apparent densities of PUMA/SiO_2_ composites were determined by measuring the dimensions and mass of the bending specimens for various SiO_2_ contents. The dimensions of the bending specimen were measured using a digital Vernier caliper with a resolution of 0.01 mm (H500-20, Mitutoyo Co., Kanagawa, Japan). The mass of the specimen was measured using a digital scale with a resolution of 0.01 g (CJ-320E, CAS Co., Ltd., Seoul, Republic of Korea). 

To analyze the dispersion characteristics of SiO_2_ particles in the UDMA resin, a field-emission type scanning electron microscopy (SEM-Vega3, Tescan, Brno, Czech Republic) was employed with a magnification of 3000. The specimens for the SEM analyses were obtained from the broken faces of the tensile specimens.

For the analysis of the chemical compositions of PUMA/SiO_2_ composites with varying SiO_2_ contents, Fourier transform infrared spectroscopy (FT-IR) was carried out. Infrared spectra were acquired using an FT-IR spectrometer (Cary 630 FTIR, Agilent Technologies, Santa Clara, CA, USA) equipped with a diamond attenuated total reflectance (ATR) accessory.

## 3. Results and Discussion

### 3.1. Additive Manufacturing of PUMA/SiO_2_ Composites

For the basic characterization of additively manufactured PUMA/SiO_2_ composites, bending and tensile specimens were fabricated using the DLP-type AM process with various SiO_2_ contents. In the AM process, the vat temperature and postcuring time were set to 35 °C and 150 s, respectively. Table 1 presents detailed information on the printed bending specimens, including the measured volume, mass, and density values. The measured density values of the PUMA/SiO_2_ composites increase with the rise in SiO_2_ content, consistent with the higher density of SiO_2_ powders (2.07 g/cm^3^).

**Figure 2:**
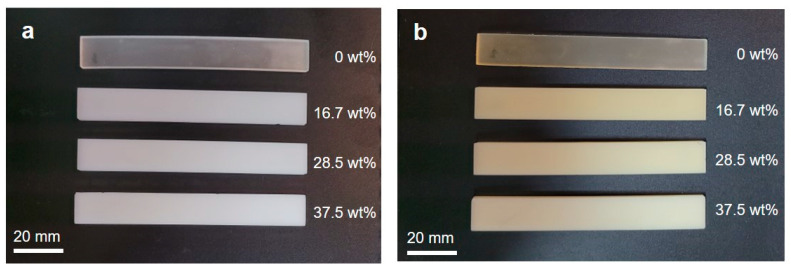
Photographs of additively manufactured PUMA/SiO_2_ composites with different SiO_2_ contents: (**a**) as-printed specimens without postcuring and (**b**) additionally cured specimens after 150 s postcuring.

Figure 2 displays photographs of the additively manufactured bending specimens. To examine the effect of photocuring on the appearance, as-printed specimens without postcuring are illustrated in Figure 2a. Whereas the PUMA sample (i.e., 0 wt%) is partially transparent, the PUMA/SiO_2_ composites containing SiO_2_ particles appear white, signifying that the inclusion of SiO_2_ particles diminishes the transparency of the PUMA photopolymer. Figure 2b shows additively manufactured bending specimens after 150 s postcuring, revealing a color shift from white to beige. This observation suggests that additional photocuring induces a color change in PUMA/SiO_2_ composites.

**Figure 3:**
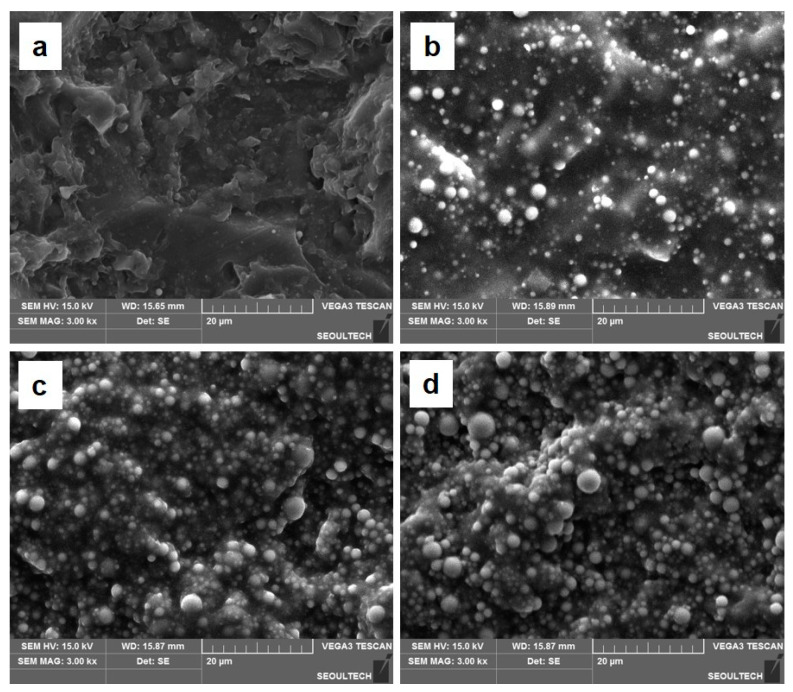
SEM images of the PUMA/SiO_2_ composites with different SiO_2_ contents: (**a**) 0 wt%, (**b**) 16.7 wt%, (**c**) 28.5 wt%, and (**d**) 37.5 wt%.

Figure 3a–d represent the SEM images of the PUMA/SiO_2_ composites with SiO_2_ contents of 0, 16.7, 28.5, and 37.5 wt%, respectively. While Figure 3c,d exhibit uniform dispersions of SiO_2_ particles, Figure 3b shows relatively non-uniform dispersion compared to the other composites. This indicates that a low SiO_2_ content, specifically 16.7 wt%, as shown in Figure 3b, induces a non-uniform particle dispersion during the blending of SiO_2_ particles and subsequent printing processes.

### 3.2. Rheological Test Results

**Figure 4:**
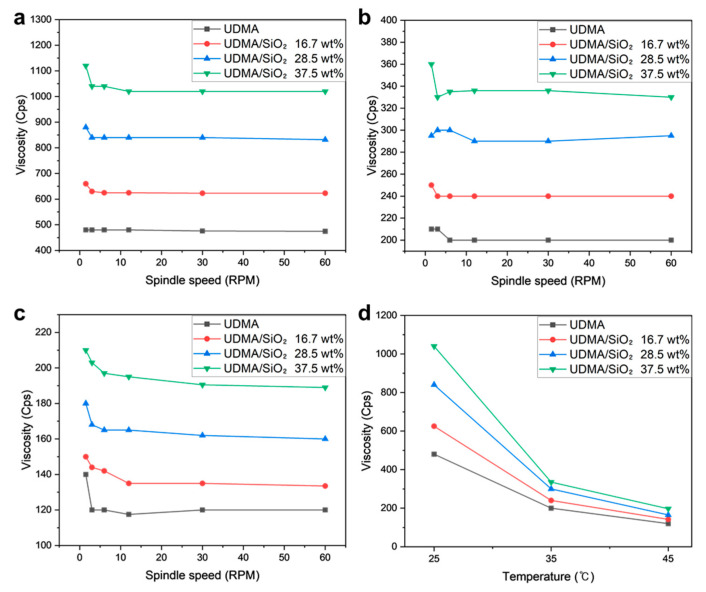
Rheological test results of additively manufactured UDMA/SiO_2_ composites under various vat temperatures: (**a**) 25 °C, (**b**) 35 °C, (**c**) 45 °C, and (**d**) comparison of viscosity value at a rotational speed of 60 rpm.

Figure 4 illustrates the viscosity profiles of the UDMA/SiO_2_ composite resins with variations in spindle speed and resin temperature. The viscosity of the UDMA/SiO_2_ composite resin exhibited an upward trend with escalating SiO_2_ weight percentage, as delineated in Figure 4a–c. Notably, the viscosity of the UDMA/SiO_2_ composite resin, with SiO_2_ constituting up to 37.5 wt%, demonstrated minimal variation in response to changes in spindle speed. This observation is indicative of a prevailing Newtonian behavior [46].

Figure 4d presents the viscosity profiles of various composite resins in response to a temperature increase. The viscosity of the composite resin exhibited a decline with rising temperatures from 25 °C to 45 °C. Notably, the viscosity of UDMA/SiO_2_ resin with higher SiO_2_ contents dramatically decreased from 1040 to 197 cps, in comparison to the UDMA resin. In the context of DLP printing, the viscosity of the composite resin is a critical parameter. Accordingly, excessively high viscosity has the potential to hinder the proper spreading of the resin after UV curing in the resin vat, leading to the formation of void spaces, and, ultimately, printing failure. It is worth highlighting that all the composite resins investigated in this study remained printable; the viscosity values for all UDMA/SiO_2_ resin formulations were below 1140 cps, which is considered relatively low viscosity within the context of DLP printing systems [47]. This ensures that the composite resins maintain suitable flow characteristics for effective printing without encountering issues related to excessive viscosity.

### 3.3. Mechanical Test Results

#### 3.3.1. Hardness Test Results

**Figure 5:**
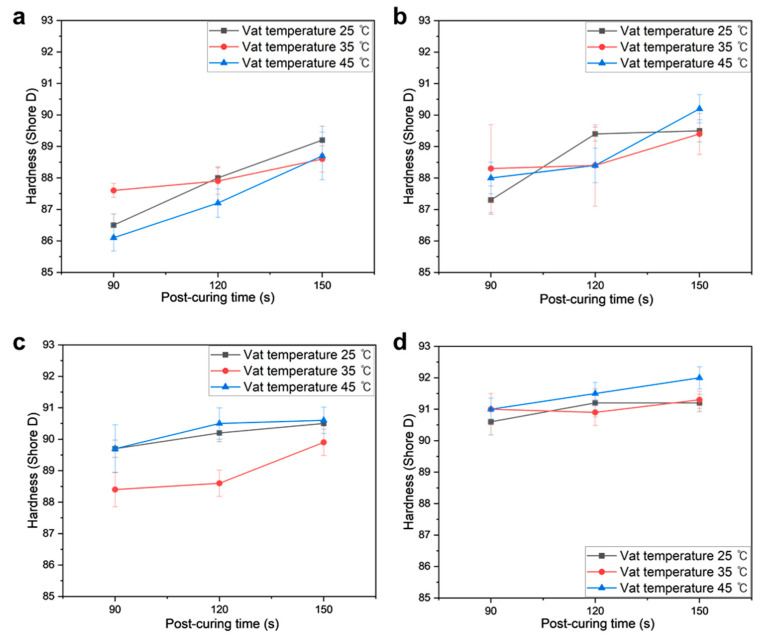
Hardness values of additively manufactured PUMA/SiO_2_ composites with different SiO_2_ contents: (**a**) 0 wt%, (**b**) 16.7 wt%, (**c**) 28.5 wt%, and (**d**) 37.5 wt%.

Figure 5 illustrates the measured hardness values of additively manufactured PUMA/SiO_2_ composites at different SiO_2_ contents. In this context, 0 wt% signifies the PUMA sample without the inclusion of SiO_2_, and each graph depicts hardness values with different vat temperatures and postcuring times. Generally, the hardness of PUMA (i.e., 0 wt% case) exhibits an increasing trend within the range of 86D to 89D, corresponding to elevated vat temperature and postcuring time. In the case of PUMA/SiO_2_ composites, hardness values increased within the range of 87D to 92D as the SiO_2_ content increased. Notably, PUMA/SiO_2_ composites with a 37.5 wt% SiO_2_ composition exhibit superior hardness values between 90D and 92D with less sensitive responses to the vat temperature and postcuring time. These findings imply that a higher SiO_2_ content imparts reduced sensitivity of hardness to variations in processing conditions. Conversely, lower SiO_2_ contents necessitate more extended postcuring times, exceeding 120 s, to achieve optimal hardness characteristics.

#### 3.3.2. Bending Test Results

**Figure 6:**
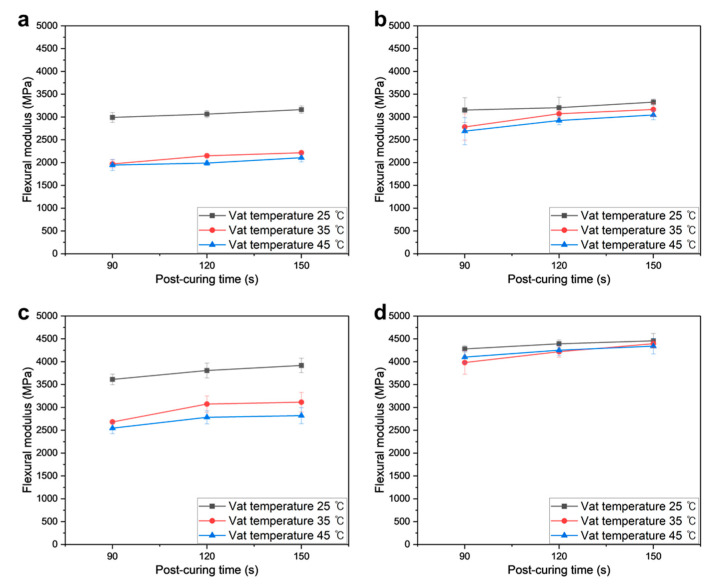
Flexural moduli of additively manufactured PUMA/SiO_2_ composites with different SiO_2_ contents: (**a**) 0 wt%, (**b**) 16.7 wt%, (**c**) 28.5 wt%, and (**d**) 37.5 wt%.

Figure 6 depicts the measured flexural modulus values of PUMA/SiO_2_ composites across varying SiO_2_ contents. Generally, an increasing trend is observed in the flexural modulus with higher SiO_2_ contents. While the postcuring time shows a positive influence, the magnitude of improvement is relatively modest compared to other parameters. In contrast, the vat temperature exerts a notable negative impact on the flexural modulus, with all specimens exhibiting the highest values when subjected to a 25 °C temperature. Notably, PUMA/SiO_2_ composites with a 37.5 wt% SiO_2_ composition exhibit the lowest deviation for different vat temperatures, ranging between 3983 and 4459 MPa, as depicted in Figure 6d. Compared to the results of the PUMA sample in Figure 6a, which fall between 1947 and 3163 MPa, these findings imply that a higher SiO_2_ content imparts an enhanced flexural modulus with reduced sensitivity to variations in processing conditions.

**Figure 7:**
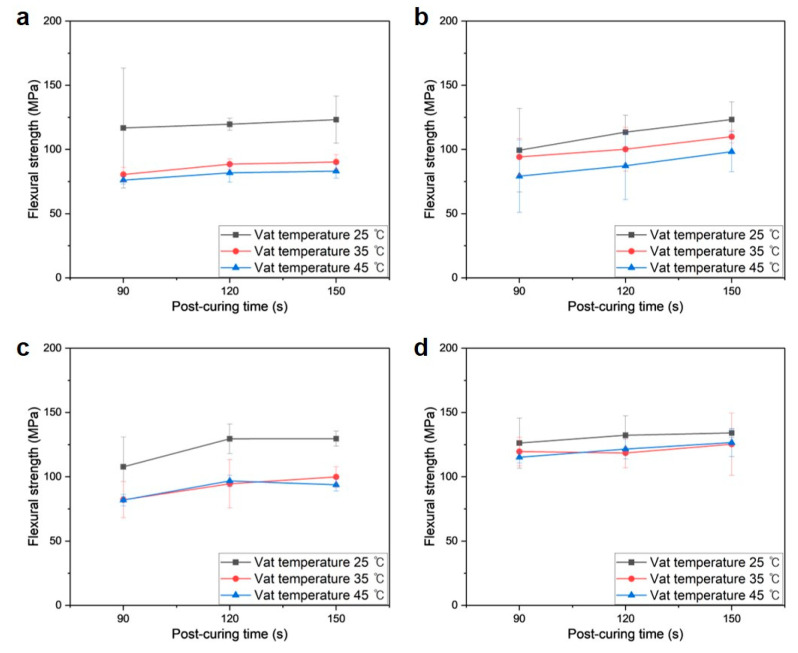
Flexural strengths of additively manufactured PUMA/SiO_2_ composites with different SiO_2_ contents: (**a**) 0 wt%, (**b**) 16.7 wt%, (**c**) 28.5 wt%, and (**d**) 37.5 wt%.

Figure 7 presents the measured flexural strengths of PUMA/SiO_2_ composites across varying SiO_2_ contents. Overall, the flexural strength exhibits a subtle increasing trend with higher SiO_2_ content and postcuring time. Similar to the observations in Figure 7, the vat temperature demonstrates a negative influence on flexural strength, with the specimens achieving the highest values at a 25ºC vat temperature. This degeneration is particularly pronounced in the case of the PUMA sample, as highlighted in Figure 7a. Despite the positive impact of higher vat temperatures on viscosity improvement, this degradation in both flexural modulus and strength is attributed to increased viscosity, causing non-uniform dispersion of SiO_2_ powders in the UDMA resin and resultant stress concentration. In contrast, PUMA/SiO_2_ composites with a 37.5 wt% SiO_2_ composition exhibit the lowest deviation across different vat temperatures (Figure 7d), indicating that the 37.5 wt% SiO_2_ content ensures the highest flexural strength with the minimal sensitivity to vat temperature variation.

#### 3.3.3. Tensile Test Results

**Figure 8:**
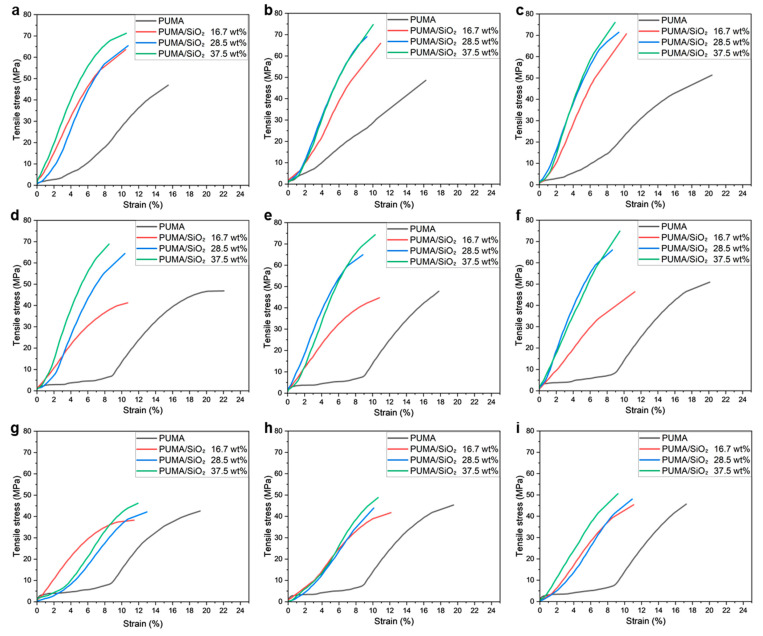
Tensile stress–strain curves of additively manufactured PUMA/SiO_2_ composites under various printing conditions: (**a**) *T_v_*_at_ = 25 °C, *t_cure_* = 90 s, (**b**) *T_v_*_at_ = 25 °C, *t_cure_* = 120 s, (**c**) *T_v_*_at_ = 25 °C, *t_cure_* = 150 s, (**d**) *T_v_*_at_ = 35 °C, *t_cure_* = 90 s, (**e**) *T_v_*_at_ = 35 °C, *t_cure_* = 120 s, (**f**) *T_v_*_at_ = 35 °C, *t_cure_* = 150 s, (**g**) *T_v_*_at_ = 45 °C, *t_cure_* = 90 s, (**h**) *T_v_*_at_ = 45 °C, *t_cure_* = 120 s, and (**i**) *T_v_*_at_ = 45 °C, *t_cure_* = 150 s.

Figure 8a–i depict the tensile stress–strain curves of various PUMA/SiO_2_ composites, with variations in the vat temperature (*T_v_*_at_) and postcuring time (*t_cure_*). In general, tensile strengths exhibit an increasing trend with rising SiO_2_ contents, while the elongation shows the opposite trend. This indicates that the inclusion of SiO_2_ particles increases the brittleness of the composites. Between the two printing parameters, the vat temperature yields more sensitive results than the postcuring time. It is noteworthy that the overall tensile strengths decreased when the vat temperature was set to 45 °C. 

The PUMA/SiO_2_ composites with 28.5 and 37.5 wt% SiO_2_ compositions exhibit nearly linear elastic behaviors, whereas the neat PUMA sample demonstrates a viscoelastic deformation behavior. Notably, the PUMA/SiO_2_ composites with a 16.7 wt% SiO_2_ composition display viscoelastic behaviors, and the tensile strengths of some cases even fall below those of the PUMA samples, as shown in Figure 8g–i. These cases correspond to situations where the vat temperature was higher than 35 °C. Considering the reduction in viscosity under those temperature conditions, the low inclusion of SiO_2_ particles leads to non-uniform dispersion, thereby resulting in the deterioration of the mechanical properties of the composite material.

**Figure 9:**
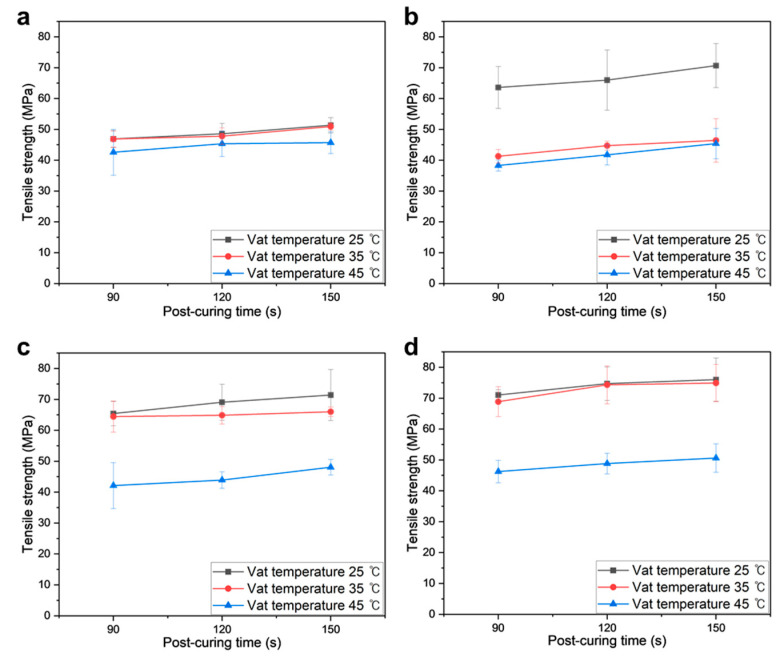
Tensile strengths of additively manufactured PUMA/SiO_2_ composites with different SiO_2_ contents: (**a**) 0 wt%, (**b**) 16.7 wt%, (**c**) 28.5 wt%, and (**d**) 37.5 wt%.

Figure 9 presents the resulting tensile strengths of PUMA/SiO_2_ composites. For the PUMA sample, the vat temperature exhibits a negative effect, while the postcuring time yields a positive impact on strength. Therefore, it is recommended to maintain a vat temperature below 35 °C and a postcuring time exceeding 120 s to enhance tensile strength. This effect becomes more pronounced with the inclusion of SiO_2_ particles. As depicted in Figure 9b, the 16.7 wt% PUMA/SiO_2_ composite at a 25 °C vat temperature demonstrates enhanced strengths, exceeding 63 MPa, compared to other temperature cases with strengths below 46 MPa. Similar strength degradations are observed in the cases of 28.5 wt% and 37.5 wt% composites under the 45 °C vat temperature condition. The decline in strength for PUMA/SiO_2_ composites under high vat temperatures can be attributed to increased viscosity during the AM process. That is, the ceramic particles in the UDMA resin may be distributed non-uniformly, leading to stress concentration. Based on these findings, to obtain high tensile strength, the SiO_2_ content is recommended to be maintained above 28.5 wt%, and the vat temperature is recommended to be maintained below 35 °C.

### 3.4. Thermal Test Results

#### 3.4.1. Thermogravimetric Analysis

**Figure 10:**
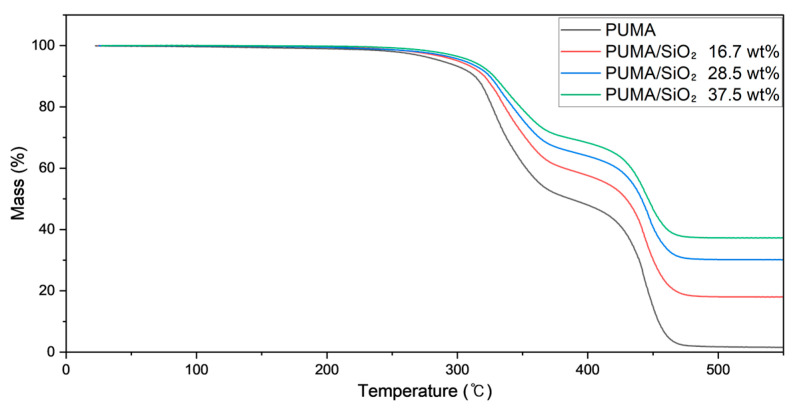
TGA curves of additively manufactured PUMA/SiO_2_ composites with different SiO_2_ contents.

Figure 10 presents the TGA scan results for PUMA/SiO_2_ composites with varying SiO_2_ contents, revealing a two-step thermal degradation process. The initial degradation occurs at 329 °C, attributed to end-chain scission involving vinylidene end groups in the UV-cured polymer. Subsequently, a second degradation step is observed at 447 °C, induced by random scission at head-to-head linkages [48,49].

The heating process extended up to 550 °C, guided by the weight retention profile exhibiting a plateau after 480 °C, attributed to the retention of SiO_2_ weight [50]. The weight retention at 550 °C allows calculation of the SiO_2_ composition in each specimen, yielding values of 18.0%, 30.2%, and 37.5%, closely aligning with the theoretical compositions of 16.7%, 28.5%, and 37.5%, respectively. Notably, deviations between theoretical and experimental data decrease as the SiO_2_ contents increase. This observation can be attributed to the order of dispersion of SiO_2_ particles; a larger quantity of SiO_2_ particles ensures a more uniform dispersion during the AM process, thereby contributing to improvements in mechanical properties, as discussed in the previous sections.

#### 3.4.2. Thermal Diffusivity Test 

Table 2 provides detailed results of thermal diffusivity tests for the PUMA/SiO_2_ composites with varying SiO_2_ contents. The thermal diffusivity, conductivity, and specific heat of the PUMA sample were measured to be 0.118 mm^2^/s, 0.194 W/m-K, and 1.435 J/g-K, respectively. As the SiO_2_ content increases, thermal diffusivity and conductivity increase, while the specific heat decreases. This can be attributed to the intrinsic thermal properties of the SiO_2_ particle, which exhibits lower thermal diffusivity (0.87 mm^2^/s) and conductivity (1.43 W/m-K) but higher specific heat (0.71 J/g-K) compared to the PUMA samples.

### 3.5. FT-IR Test Results

**Figure 11:**
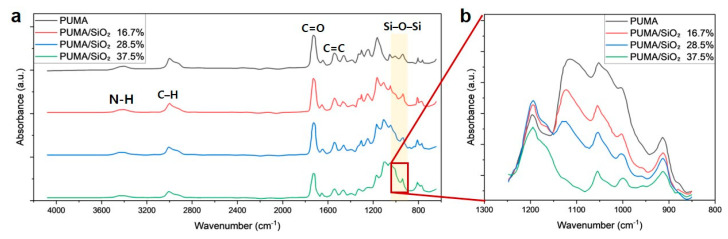
FT-IR analysis results: (**a**) FT-IR spectra of the PUMA/SiO_2_ composites with different SiO_2_ contents, (**b**) magnification of select peaks in the range between 850 and 1250 cm^−1^.

Figure 11a presents the FT-IR characterization results of PUMA/SiO_2_ composites with varying SiO_2_ contents. The FT-IR spectra of the four PUMA/SiO_2_ composites exhibited absorption peaks corresponding to the stretching vibrations of the N-H group at 3350 cm^−1^, the C-H group at 2960 cm^−1^, the C=O carbonyl group at 1730 cm^−1^, and the aliphatic C=C group at 1638 cm^−1^ [51]. The presence of SiO_2_ was confirmed by the absorption peak for Si-O-Si asymmetric stretching vibration between 1000 cm^−1^ and 1100 cm^−1^ [52]. Figure 11b shows a magnified view in the range between 850 and 1250 cm^−1^, highlighting that the intensity of the Si-O-Si band became more pronounced with an increase in SiO_2_ content.

## 4. Conclusions

This study investigates the DLP-type AM of ceramic composites with a focus on enhancing mechanical properties. A composite resin was formulated by blending UDMA photocurable resin and microscale SiO_2_ powders, and PUMA/SiO_2_ composites with varied SiO_2_ contents (16.7, 28.5, and 37.5 wt%) were then manufactured through the DLP-type AM and subsequent postcuring processes. The mechanical and thermal properties of these composites were systematically analyzed, aided by chemical analysis, considering variations in vat temperature and postcuring time. The key findings from these analyses are summarized as follows:

- Surface hardness: PUMA/SiO_2_ composites with a 37.5 wt% SiO_2_ composition exhibited the highest hardness values with less sensitivity to processing conditions. In contrast, composites with lower SiO_2_ contents displayed inferior hardness and larger deviations.
- Flexural properties: Elevated vat temperatures (45 °C) were found to degrade both flexural modulus and strength. In contrast, composites with a SiO_2_ content of 37.5 wt% demonstrated the highest flexural modulus, surpassing twice that of the pure PUMA sample.
- Tensile properties: PUMA/SiO_2_ composites with high SiO_2_ contents, exceeding 28.5 wt%, exhibited linear elastic behavior with improved tensile strength while the pure PUMA sample exhibited viscoelastic behavior. Notably, maintaining a low vat temperature (less than 35 °C) ensured high tensile strength for PUMA/SiO_2_ composites.
- Thermal properties: The inclusion of SiO_2_ particles increased thermal conductivity and diffusivity, revealing that a 37.5 wt% SiO_2_ composition exhibited the highest values. TGA tests indicated that a larger quantity of SiO_2_ particles ensured a more uniform dispersion during the AM process, contributing to improvements in mechanical properties.

A comprehensive synthesis of these findings leads to guidelines for the optimal usage of PUMA/SiO_2_ composites, emphasizing the importance of a high SiO_2_ content and a low vat temperature to enhance mechanical properties and ensure thermal stability. More specifically, the composition with 37.5 wt% SiO_2_ exhibited optimal performance, showing enhanced mechanical properties and thermal stability with the minimal sensitivity to processing conditions. These guidelines are expected to inform future endeavors in developing functional composite parts with enhanced mechanical properties and thermal stability.

## Figures and Tables

**Table 1:** Comparison of volume, mass, and density values of PUMA/SiO_2_ composites.

SiO_2_ Content	Volume (cm^3^)	Mass (g)	Density (g/cm^3^)
0 wt%	3.174 ± 0.009	3.483 ± 0.005	1.097 ± 0.002
16.7 wt%	3.168 ± 0.013	3.748 ± 0.014	1.183 ± 0.002
28.5 wt%	3.138 ± 0.138	4.045 ± 0.006	1.289 ± 0.006
37.5 wt%	3.170 ± 0.006	4.204 ± 0.019	1.326 ± 0.004

**Table 2:** Comparison of thermal properties of additively manufactured PUMA/SiO_2_ composites with different SiO_2_ contents.

SiO_2_ Content	*α* (mm^2^/s)	*k* (W/m-K)	*C_p_* (J/g-K)
0 wt%	0.118	0.194	1.435
16.7 wt%	0.147	0.236	1.279
28.5 wt%	0.156	0.256	1.252
37.5 wt%	0.173	0.281	1.174

## Data Availability

Data are contained within the article.

## References

[1] Filser F., Kocher P., Gauckler L.J. (2003). Net-shaping of ceramic components by direct ceramic machining. Assem. Autom..

[2] Ayode O.T., Ugochukwu O.P., Chen G., Li Y., Onyeka O.M., Li S. (2020). Advanced ceramic components: Materials, fabrication, and applications. J. Ind. Eng. Chem..

[3] Robinson S.K., Paul M.R. (2001). Debinding and sintering solutions for metals and ceramics. Met. Powder Rep..

[4] Chen Z., Li Z., Li J. (2019). 3D printing of ceramics: A review. J. Eur. Ceram. Soc..

[5] Zocca A., Colombo P., Gomes C.M., Günster J. (2015). Additive manufacturing of ceramics: Issues, potentialities, and opportunities. J. Am. Ceram. Soc..

[6] Moon J., Grau J.E., Knezevic V., Cima M.J., Sachs E.M. (2002). Ink-jet printing of binders for ceramic components. J. Am. Ceram. Soc..

[7] Zhang W., Melcher R., Travitzky N., Bordia R.K., Greil P. (2009). Three-dimensional printing of complex-shaped alumina/glass composites. Adv. Eng. Mater..

[8] Travitzky N., Bonet A., Dermeik B. (2014). Additive manufacturing of ceramic-based materials. Adv. Eng. Mater..

[9] Chavez L.A., Ibave P., Wilburn B. (2020). The influence of printing parameters, post-processing, and testing conditions on the properties of binder jetting additive manufactured functional ceramics. Ceramics.

[10] Tang H.H., Chiu M.L., Yen H.C. (2011). Slurry-based selective laser sintering of polymer-coated ceramic powders to fabricate high strength alumina parts. J. Eur. Ceram. Soc..

[11] Shahzad K., Deckers J., Zhang Z., Kruth J.P., Vleugels J. (2014). Additive manufacturing of zirconia parts by indirect selective laser sintering. J. Eur. Ceram. Soc..

[12] Deckers J.P., Shahzad K., Cardon L., Rombouts M., Vleugels J., Kruth J.P. (2016). Shaping ceramics through indirect selective laser sintering. Rapid Prototyp. J..

[13] Kumar S. (2009). Manufacturing of WC–Co moulds using SLS machine. J. Mater. Process. Technol..

[14] Mohamed O.A., Masood S.H., Bhowmik J.L. (2015). Optimization of fused deposition modeling process parameters: A review of current research and future prospects. Adv. Manuf..

[15] Allahverdi M., Danforth S.C., Jafari M., Safari A. (2001). Processing of advanced electroceramic components by fused deposition technique. J. Eur. Ceram. Soc..

[16] Iyer S., McIntosh J., Bandyopadhyay A., Langrana N., Safari A., Danforth S., Clancy R., Gasdaska C., Whalen P. (2008). Microstructural characterization and mechanical properties of Si_3_N_4_ formed by fused deposition of ceramics. Int. J. Appl. Ceram. Technol..

[17] Khatri B., Lappe K., Habedank M., Mueller T., Megnin C., Hanemann T. (2018). Fused deposition modeling of ABS-Barium Titanate composites: A simple route towards tailored dielectric devices. Polymers.

[18] Smirnov A., Terekhina S., Tarasova T., Hattali L., Grigoriev S. (2023). From the development of low-cost filament to 3D printing ceramic parts obtained by fused filament fabrication. Int. J. Adv. Manuf. Technol..

[19] Rashid A., Ahmed W., Khalid M.Y., Koç M. (2021). Vat photopolymerization of polymers and polymer composites: Processes and applications. Addit. Manuf..

[20] Quan H., Zhang T., Xu H., Luo S., Nie J., Zhu X. (2020). Photo-curing 3D printing technique and its challenges. Bioact. Mater..

[21] Bae C.J., Halloran J.W. (2011). Integrally cored ceramic mold fabricated by ceramic stereolithography. Int. J. Appl. Ceram. Technol..

[22] Kirihara S., Niki T. (2015). Three-dimensional stereolithography of alumina photonic crystals for terahertz wave localization. Int. J. Appl. Ceram. Technol..

[23] Lian Q., Sui W., Wu X., Yang F., Yang S. (2018). Additive manufacturing of ZrO_2_ ceramic dental bridges by stereolithography. Rapid Prototyp. J..

[24] Stampfl J., Schwentenwein M., Homa J., Prinz F.B. (2023). Lithography-based additive manufacturing of ceramics: Materials, applications and perspectives. MRS Commun..

[25] Chaudhary R., Fabbri P., Leoni E., Mazzanti F., Akbari R., Antonini C. (2022). Additive manufacturing by digital light processing: A review. Prog. Addit. Manuf..

[26] Wei Y., Zhao D., Cao Q. (2020). Stereolithography-based additive manufacturing of high-performance osteoinductive calcium phosphate ceramics by a digital light-processing system. ACS Biomater. Sci. Eng..

[27] Terner M. (2019). Innovative 3D-manufacturing of complex ceramic parts by means of commercial digital light processing apparatus. J. Weld. Join..

[28] Chaudhary R., Akbari R., Antonini C. (2023). Rational design and characterization of materials for optimized additive manufacturing by digital light processing. Polymers.

[29] Kim T.Y., Park S.H., Park K. (2021). Development of functionally graded metamaterial using selective polymerization via digital light processing additive manufacturing. Addit. Manuf..

[30] Truxova V., Safka J., Seidl M., Kovalenko I., Volesky L., Ackermann M. (2020). Ceramic 3d printing: Comparison of SLA and DLP technologies. MM Sci. J..

[31] Goldberg M., Obolkina T., Smirnov S. (2020). The influence of Co additive on the sintering, mechanical properties, cytocompatibility, and digital light processing based stereolithography of 3Y-TZP-5Al_2_O_3_ ceramics. Materials.

[32] Zhao W., Wang C., Xing B., SShe M., Zhao Z. (2020). Mechanical properties of zirconia octet truss structures fabricated by DLP 3D printing. Mater. Res. Express.

[33] He C., Ma C., Li X. (2020). Polymer-derived SiOC ceramic lattice with thick struts prepared by digital light processing. Addit. Manuf..

[34] Huang R.J., Jiang Q.G., Wu H.D. (2019). Fabrication of complex shaped ceramic parts with surface-oxidized Si_3_N_4_ powder via digital light processing based stereolithography method. Ceram. Int..

[35] Schmidt J., Colombo P. (2018). Digital light processing of ceramic components from polysiloxanes. J. Eur. Ceram. Soc..

[36] Sun Y., Li M., Jiang Y. (2021). High-quality translucent alumina ceramic through digital light processing stereolithography method. Adv. Eng. Mater..

[37] Zhang D., Yang Y., Rao W.F., Zhang D., Yang Y., Rao W.F. (2023). Parameter optimization for printing barium titanate piezoelectric ceramics through digital light processing. Micromachines.

[38] Corcione C.E., Greco A., Montagna F., Licciulli A., Maffezzoli A. (2005). Silica moulds built by stereolithography. J. Mater. Sci..

[39] Chartier T., Badev A., Abouliatim Y., Lebaudy P., Lecamp L. (2012). Stereolithography process: Influence of the rheology of silica suspensions and of the medium on polymerization kinetics—Cured depth and width. J. Eur. Ceram. Soc..

[40] Zhou W., Li D., Chen Z. (2011). The influence of ingredients of silica suspensions and laser exposure on UV curing behavior of aqueous ceramic suspensions in stereolithography. Int. J. Adv. Manuf. Technol..

[41] Kićević D., Gašić M., Marković D. (1996). A statistical analysis of the influence of processing conditions on the properties of fused silica. J. Eur. Ceram. Soc..

[42] Dehghani P., Soleimani F. (2021). Effect of cristobalite content on physical, dielectric constant, and bending strength of fused silica ceramics formed by slip casting method. Adv. Ceram. Prog..

[43] (2019). Plastics—Determination of Flexural Properties.

[44] (2017). Standard Test Method for Tensile Properties of Plastics.

[45] (2022). Standard Test Method for Thermal Diffusivity by the Flash Method.

[46] Stefan E., Didriksen T., Sunde T.O., Fontaine M.L., Ræder H., Rørvik P.M. (2023). Effects of powder properties on the 3D printing of BaTiO_3_ ceramic resins by stereolithography. Prog. Addit. Manuf..

[47] Lin C.H., Lin Y.M., Lai Y.L., Lee S.Y. (2020). Mechanical properties, accuracy, and cytotoxicity of UV-polymerized 3D printing resins composed of Bis-EMA, UDMA, and TEGDMA. J. Prosthet. Dent..

[48] Bakar A.A., Zainuddin M.Z., Abdullah S.M. (2022). The 3D printability and mechanical properties of polyhydroxybutyrate (PHB) as additives in urethane dimethacrylate (UDMA) blends polymer for medical application. Polymers.

[49] Achilias D.S., Karabela M.M., Sideridou I.D. (2008). Thermal degradation of light-cured dimethacrylate resins: Part I. Isoconversional kinetic analysis. Thermochim. Acta.

[50] Fantino E., Chiappone A., Roppolo I. (2016). 3D printing of conductive complex structures with in situ generation of silver nanoparticles. Adv. Mater..

[51] Du M., Zheng Y. (2008). Degree of conversion and mechanical properties studies of UDMA based materials for producing dental posts. Polym. Compos..

[52] Ellerbrock R., Stein M., Schaller J. (2022). Comparing amorphous silica, short-range-ordered silicates and silicic acid species by FTIR. Sci. Rep..

